# Hypercortisolism and primary aldosteronism caused by bilateral adrenocortical adenomas: a case report

**DOI:** 10.1186/s12902-019-0395-y

**Published:** 2019-06-17

**Authors:** Kaiyun Ren, Jia Wei, Qilin Liu, Yuchun Zhu, Nianwei Wu, Ying Tang, Qianrui Li, Qianying Zhang, Yerong Yu, Zhenmei An, Jing Chen, Jianwei Li

**Affiliations:** 1 Department of Endocrinology and Metabolism, West China Hospital, Sichuan University, Chengdu, 610041 China; 2 Department of Urology, West China Hospital, Sichuan University, Chengdu, 610041 China; 3 Department of Pathology, West China Hospital, Sichuan University, Chengdu, 610041 China; 4 Department of Pathology, Immunology, and Laboratory Medicine, University of Florida, Gainesville, FL 32610 USA

**Keywords:** Bilateral adrenal adenomas, Primary aldosteronism, Hypercortisolism, Adrenal venous sampling, Histopathology

## Abstract

**Background:**

Co-existing Cushing’s syndrome and primary aldosteronism caused by bilateral adrenocortical adenomas, secreting cortisol and aldosterone, respectively, have rarely been reported. Precise diagnosis and management of this disorder constitute a challenge to clinicians due to its atypical clinical manifestations and laboratory findings.

**Case presentation:**

We here report a Chinese male patient with co-existing Cushing’s syndrome and primary aldosteronism caused by bilateral adrenocortical adenomas, who complained of intermittent muscle weakness for over 3 years. Computed tomography scans revealed bilateral adrenal masses. Undetectable ACTH and unsuppressed cortisol levels by dexamethasone suggested ACTH-independent Cushing’s syndrome. Elevated aldosterone to renin ratio and unsuppressed plasma aldosterone concentration after saline infusion test suggested primary aldosteronism. Adrenal venous sampling adjusted by plasma epinephrine revealed hypersecretion of cortisol from the left adrenal mass and of aldosterone from the right one. A sequential bilateral laparoscopic adrenalectomy was performed. The cortisol level was normalized after partial left adrenalectomy and the aldosterone level was normalized after subsequent partial right adrenalectomy. Histopathological evaluation of the resected surgical specimens, including immunohistochemical staining for steroidogenic enzymes, revealed a left cortisol-producing adenoma and a right aldosterone-producing adenoma. The patient’s symptoms and laboratory findings resolved after sequential adrenalectomy without any pharmacological treatment.

**Conclusions:**

Adrenal venous sampling is essential in diagnosing bilateral functional adrenocortical adenomas prior to surgery. Proper interpretation of the laboratory findings is particularly important in these patients. Immunohistochemistry may be a valuable tool to identify aldosterone/cortisol-producing lesions and to validate the clinical diagnosis.

**Electronic supplementary material:**

The online version of this article (10.1186/s12902-019-0395-y) contains supplementary material, which is available to authorized users.

## Background

The human adult adrenal cortex is composed of the zona glomerulosa (ZG), zona fasciculata (ZF), and zona reticularis (ZR), which are responsible for production of mineralocorticoids, glucocorticoids, and adrenal androgens, respectively [1]. Primary aldosteronism (PA) and Cushing’s syndrome (CS) are usually caused by autonomous over-secretion of aldosterone or cortisol, from the corresponding adrenal zones as a result of hyperplasia or tumors. Both overt and subclinical PA and CS have long-term adverse effects on multiple organs [2, 3]. Patients with concurrent hypersecretion of cortisol and aldosterone have been previously reported, and it is difficult to make the correct diagnosis in those patients [4–8], however, most of these patients had adenomas that secreted both cortisol and aldosterone simultaneously, i.e. aldosterone- and cortisol-co-secreting adrenal tumors. Patients with bilateral adenomas that independently secrete different hormones are extremely rare. Herein, we report a patient with co-existing cortisol-producing and aldosterone-producing adrenocortical adenomas, one in each adrenal gland. The diagnosis was initially made by adrenal venous sampling (AVS) and subsequently validated by postoperative pathology findings. Additionally, we reviewed similar cases reported to summarize the diagnosis and management of this disorder.

## Case presentation

A 30-year-old Chinese male was admitted to the local hospital with a history of intermittent muscle weakness for over 3 years. He was found with high blood pressure (170/118 mmHg), hypokalemia (2.0 mmol/L), normal thyroid function, and bilateral adrenal masses on computed tomography (CT). He had no history of alcohol or drug abuse, in particular, no history of steroid usage, and no family history of endocrine diseases or malignant tumors. He was treated with a temporary prescription of nifedipine, potassium chloride controlled release tablets, and was referred to our hospital for further investigation.

Upon admission to our hospital on August 11, 2016, physical examinations revealed blood pressure of 153/100 mmHg and pulse rate of 76 beats per minute. His body mass index was 29.1 kg/m^2^, height 176 cm, weight 90 kg, and waist circumference 98 cm. There were no specific findings on chest or abdominal examination and his muscle power was normal. No edema of the lower extremities was noted and he had no Cushingoid features (i.e., moon face, purple striae, or hirsutism) except slight central obesity.

**Fig. 1:**
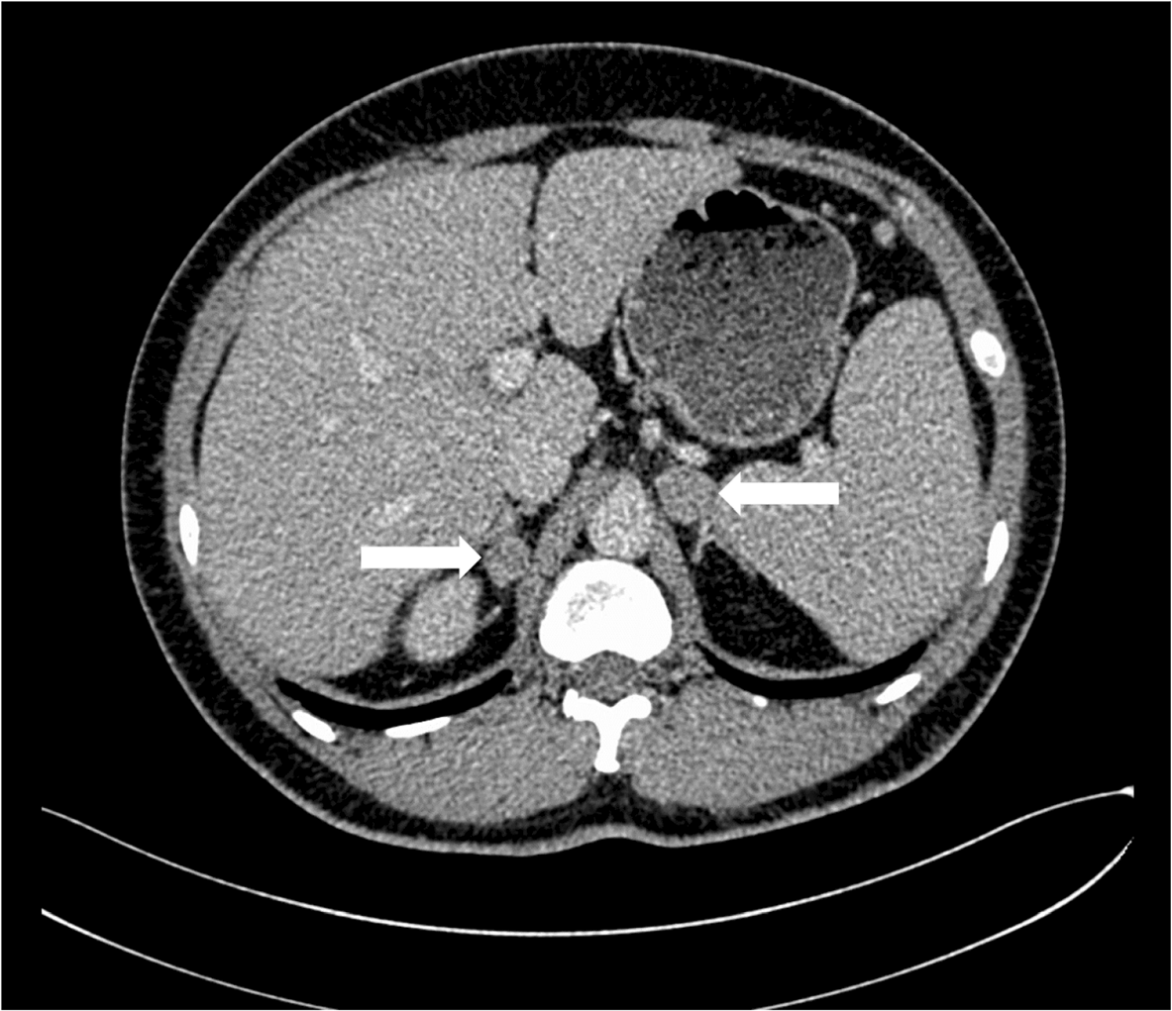
Adrenal computed tomography (CT) image showing a 19 × 14 mm right adrenal tumor and a 25 × 15 mm left adrenal tumor (arrows)

Routine laboratory tests revealed an extremely low serum potassium (2.12 mmol/L) with relatively high urinary potassium (38.66 mmol/24 h). Twenty-four-hour urinary free cortisol was 140.7 μg and 137.7 μg on two separate occasions (reference range: 20.26–127.55 μg/24 h). Detailed relevant biochemical and endocrinological findings are shown in Table 1. His aldosterone-to-renin ratio (ARR) was within normal range after discontinuation of nifedipine for more than 2 weeks, when drug-induced false-negative results were likely eliminated. Thus, further screening for PA was not performed (Table 2) [9]. In overnight and standard low-dose dexamethasone suppression tests, dexamethasone failed to suppress endogenous cortisol secretion, indicating CS (Table 3). Adrenal CT scan revealed one round, homogeneous, low-density mass in each adrenal gland. The mass on the right was 19 × 14 mm while the one on the left was 25 × 15 mm (Fig. 1). Bilateral AVS was performed to lateralize the functional side. Concentrations of plasma aldosterone and cortisol were measured in specimens from both adrenal veins (AV) and the inferior vena cava (IVC), and corrected by concentration of plasma epinephrine. As shown in Table 4, epinephrine concentrations in both AVs were approximately 10-fold higher than that in the IVC, suggesting successful adrenal venous catheterization. Notably, at > 200 ng/dL, the aldosterone concentration in the right adrenal vein was markedly higher than those in the LAV and the IVC, suggesting excess secretion of aldosterone from the right adrenal mass. Additionally, 7701 nmol/L, the cortisol concentration in LAV was 12.81-fold higher than that in the RAV and 27.4-fold higher than that in the IVC, suggesting excess secretion of cortisol from the left adrenal mass. Thus, with AVS, hypersecretion of cortisol from the left adrenal tumor and that of aldosterone from the right tumor was identified.

**Table 1:** Laboratory characteristics

	Upon admission	After left partial adrenalectomy	After right partial adrenalectomy	Reference values
WBC (10^9^/L)	7.22	6.75	–	3.5–9.5
Hb (g/L)	143	151	–	115–150
Plt (10^9^/L)	205	249	–	100–300
Glu (mmol/L)	4.06	3.98	4.95	3.9–5.9
ALT (IU/L)	45	28	52	< 50
AST (IU/L)	27	18	31	< 40
Cre (umol/L)	49.0	51.0	73.0	37.0–110.0
BUN (mmol/L)	3.90	2.50	5.20	3.13–8.17
LDL-C (mmol/L)	2.35	2.79	2.63	< 4.0
TG (mmol/L)	2.20	3.33	1.96	0.29–1.83
K (mmol/L)	2.12	1.98	4.68	3.5–5.3
Na (mmol/L)	142.9	144.0	140.0	137.0–147.0
HbA1c (%)	5.1	–	–	4.5–6.1
Plasma norepinephrine (ng/L)	180	–	–	174–357
Plasma epinephrine (ng/L)	86	–	–	60–104
Urinary norepinephrine (μg/24 h)	30.97	–	–	16.3–41.5
Urinary epinephrine (μg/24 h)	5.15	–	–	7.5–21.9
Urinary free cortisol (μg/24 h)	140.7/ 137.7	–	–	20.26–127.55
Urinary K (mmol/24 h)	38.66	–	–	–

*ALT* alanine aminotransferase, *AST* aspartate transaminase, *BUN* blood urine nitrogen, *Cre* creatinine, *Glu* glucose, *Hb* hemoglobin, *HbA1c* glycosylated hemoglobin, *K* kalium, *LDL-c* low-density lipoprotein cholesterol, *Na* natrium, *Plt* platelet, *TG* triglyceride, *WBC* white blood cell count

**Table 2:** Results of the ARR tests, saline infusion test and captopril challenge test

		PRA (ng/ml.h)	PAC (ng/dl)	ARR (ng/dl:ng/ml.h)
Before operation		2.03	25.90	12.76
After left partial adrenalectomy	Before saline infusion	0.49	34.79	71.00
	After saline infusion	0.70	26.94	38.49
	Before captopril challenge	1.89	35.38	18.72
	After captopril challenge	1.42	31.17	21.95
After right partial adrenalectomy		5.04	13.81	2.74

*PRA* plasma renin activity, *PAC* plasma aldosterone concentration, *ARR* aldosterone-to-renin ratio

**Table 3:** Results of the Dexamethasone Suppression Tests

		PTC-24 (nmol/L)	PTC-8 (nmol/L)	PTC-N8 (nmol/L)	ACTH (ng/L)
Before operation	Baseline	146	346	–	17.82
	1 mg ODMST	–	–	273	5.76
	Standard low-dose DMST	–	274	274	< 1.00
After left partial adrenalectomy	Baseline	63	305	–	38.16
	0.5 mg ODMST	29	–	32	3.10
After right partial adrenalectomy	Baseline	–	389	–	43.27

*ODMST* overnight dexamethasone suppression test, *DMST* dexamethasone suppression test, *PTC-24* plasma total cortisol at 24:00, *PTC-8* plasma total cortisol at 08:00, *PTC-N8* plasma total cortisol at next 08:00 after DMST, *ACTH* adrenocorticotropic hormone

**Table 4:** Results of adrenal venous sampling

	Aldosterone (ng/dL)	Cortisol (nmol/L)	Epinephrine (ng/L)
LAV	42.34	7701	607
RAV	>200.00	601	576
IVC	22.00	281	66
LAV: RAV ratio (left/ right)	<0.21	12.81	1.05

*AV* adrenal vein, *IVC* inferior vena cava

## Treatment and follow-up

A sequential partial adrenalectomy (laparoscopic left partial adrenalectomy followed by endoscopic right partial adrenalectomy) was performed with the agreement of the patient. Hydrocortisone was given intravenously during the left partial adrenalectomy, and was maintained at a daily dose of 150–75 mg for 5 days after the operation. The patient did not have any symptoms associated with adrenal insufficiency after the hydrocortisone supplementation discontinued. Ten days after left partial adrenalectomy, the patient’s cortisol concentrations retained normal circadian variations and 0.5 mg dexamethasone suppressed endogenous cortisol secretion (plasma total cortisol was 32 nmol/L at 08:00 the next day after 0.5 mg overnight dexamethasone suppression test), which strongly supported that the resected left adrenocortical adenoma had been producing cortisol (Table 3). The rapid recovery of cortisol circadian might be explained by his Cushing’s syndrome being mild and subclinical. However, the patient remained hypertensive (165/118 mmHg) and hypokalemia (serum potassium was 1.98 mmol/L, Table 1). Accordingly, after potassium supplement, saline infusion and captopril challenge tests were performed. The results revealed a high plasma aldosterone concentration and suppressed plasma renin activity (PRA), which supported the diagnosis of primary hyperaldosteronism (Table 2) [9]. Four days after endoscopic right partial adrenalectomy, the patient’s blood pressure and serum potassium returned normal.

**Fig. 2:**
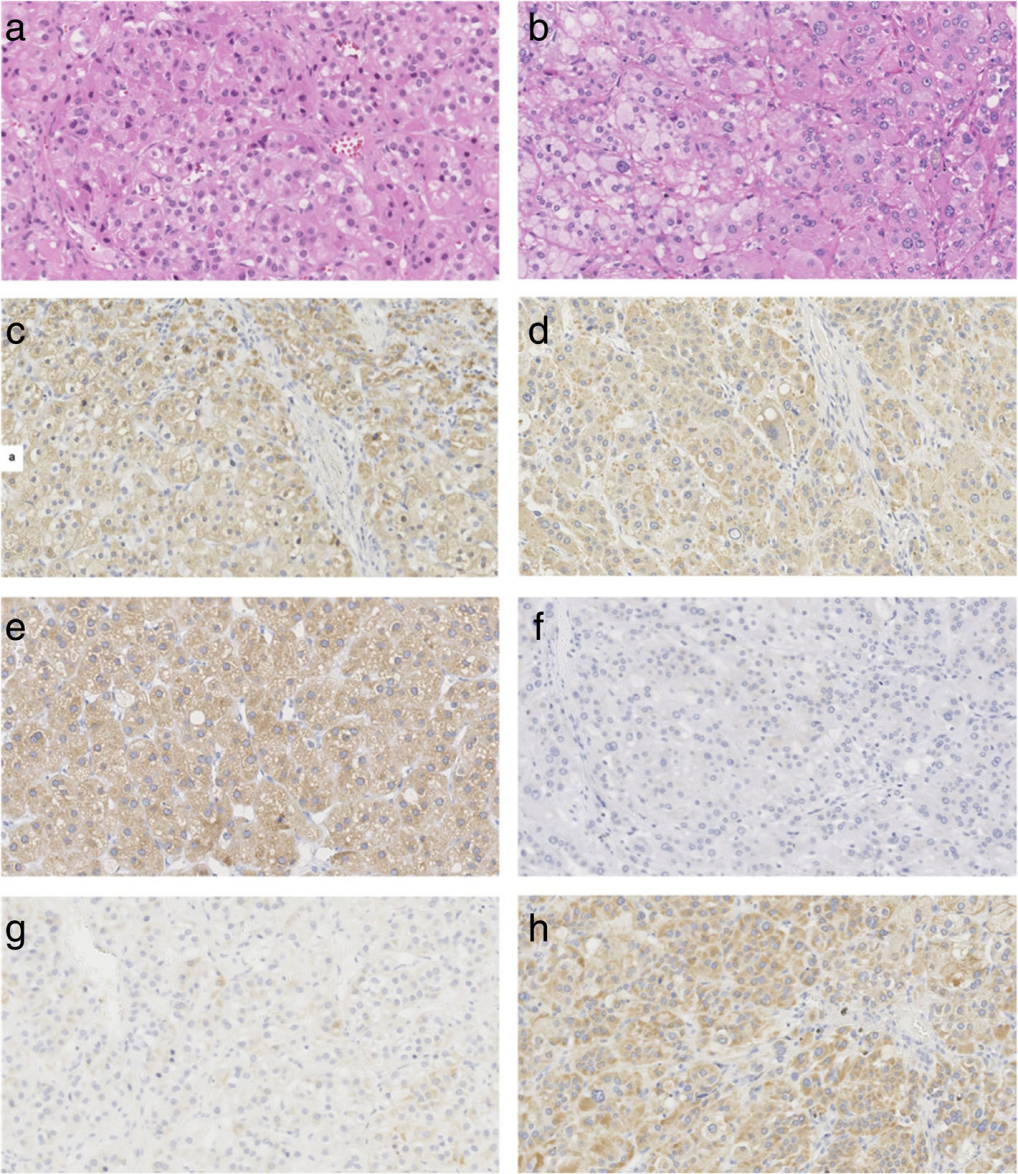
Histological and immunohistochemical findings. (a, c, e, g) Left adrenal gland. (b, d, f, h) Right adrenal gland. (**a**). Photomicrograph of left adrenal mass showing it is composed mainly of eosinophilic compact cells (H&E stain, × 200). (**b**). Photomicrograph of right adrenal mass showing it consists of both clear and compact cells, the former type predominating (H&E stain, × 200). (**c**, **d**) Immunohistochemical demonstration of 3β-HSD. (**e**, **f**) Immunohistochemical demonstration of P450c17. (**g**, **h**) Immunohistochemical demonstration of CYP11B2

Pathological examination revealed that the left adrenal mass was a 3.0 × 2.5 × 2.3 cm golden-yellow adenoma, composed mainly of eosinophilic compact cells on hematoxylin/eosin (HE) stained sections (Fig. 2a), and the right adrenal tumor was a 2.0 × 1.7 × 1.5 cm and bright yellow adenoma, consisted of both clear and compact cells, with the former cell type being dominant (Fig. 2b). To understand the function of resected masses, we further performed immuno-histochemical analysis for steroidogenic enzymes. The left mass was found with diffused presence of 17alpha-hydroxylase1 (P450c17) (Fig. 2c) and local presence of 3b-hydroxysteroid dehydrogenase (3β-HSD) (Fig. 2e) with no expression of 11beta-hydroxylase 2 (CYP11B2) (Fig. 2g). The right mass showed negative immunoreactivity to P450c17 (Fig. 2f) with increased homogenous immunoreactivity to 3β-HSD and CYP11B2 (Fig. 2d and h, respectively). These immuno-histochemical results confirmed that the patient had a co-existing right aldosterone-producing adenoma and a left cortisol-producing adrenal adenoma.

His blood pressure and serum potassium remained normal one month after the sequential partial adrenalectomy, without any medication. The laboratory findings at the last follow-up compared to those upon admission are shown in Table 1.

## Review of published reports

We searched PubMed for case reports of bilateral adenomas secreting different hormones independently till December 10, 2018, using the combinations of the following keywords: “Cushing’s syndrome”, “hypercortisolism”, “primary aldosteronism” and “bilateral adrenocortical adenoma”. All references of included reports and relevant reviews were screened manually for additional potential eligible cases. The search was limited to full-text articles published in English.

Only five cases with bilateral functioning adenomas [10–14] were found. Among reported patients, all were hypertensive, four presented with hypokalemia [10–12, 14] and three had typical Cushingoid features [10, 12, 13]. It is noteworthy that only two patients presented with all three typical characteristics, namely hypertension, hypokalemia and Cushingoid features [10, 12]. The diagnosis was made preoperatively based on results of AVS in all five patients and was confirmed by immunohistochemical staining for steroidogenic enzymes in only three [11–13]. Detailed information on each reported patients is presented in Additional file 1: Table S1, including name of first author, year of publication, country, patient characteristics (sex and age at diagnosis), symptoms, preoperative diagnostic technique, surgical operation, appearance of cut surface of tumor, and histopathological findings.

## Discussion

We here present a case of bilateral adrenal adenomas, left of which produced cortisol and right produced aldosterone, as determined by AVS and confirmed by immunohistochemical analysis. Many reported cases of co-existing hypersecretion of cortisol and aldosterone were solitary or multiple adrenocortical adenomas that secrete both aldosterone and cortisol simultaneously as reviewed by Spath et al. [4]. However, it is extremely rare reported that multiple adrenocortical adenomas located bilaterally secrete different types of hormones from each side.

The presented patient did not have typical Cushingoid features or PA, which promoted a challenge in clinical practice to establish accurate diagnosis. Our review of similar cases suggested that this atypical manifestation was common among patients with co-existing hyperaldosteronism and hypercortisolism. The lacking of overt Cushingoid clinical features might be attributable to the secretion of cortisol remaining under adrenocorticotropic hormone (ACTH) feedback control and circulating cortisol concentration being not high enough to induce overt Cushingoid features. Since PRA was measured by radioimmunoassay of generated angiotensin I, the failure of aldosterone to suppress renin, presented as a normal ARR that excluded our patient from further PA screening upon admission, might be caused by the interference from high plasma cortisol which could significantly increase plasma angiotensinogen level and consequently increase generated angiotensin I, leading to falsely increased PRA [15]. Guthrie and Kotchen reported a similar case with a unilateral adrenal adenoma producing both cortisol and aldosterone, in which renin was not suppressed by aldosterone [16], same as in this patient. Previous studies also found that the renin–angiotensin–aldosterone system was affected by plasma ACTH concentration in patients with aldosterone-producing adenomas (APAs) [17–19]. As supported by a review of 35 patients with aldosterone- and cortisol-co-secreting tumors by Spath et al., the atypical clinical manifestations and laboratory findings among these patients were likely to cause misinterpretation of diagnostic test results, if not dealt with caution [4].

Given that the coexistence of aldosterone and cortisol co-secreting adrenal tumors is not uncommon, we recommended that patients with suspected primary hyperaldosteronism should also be screened for hypercortisolism, and that a low-dose overnight dexamethasone suppression test was likely adequate for this purpose. Moreover, careful interpretation of laboratory results of the renin–angiotensin–aldosterone system is necessary, especially when secretion of endogenous ACTH is suppressed, as seen in our patient. Furthermore, for patients with bilateral adrenal cortical adenomas, if biochemical evidence suggests co-existing of hypercortisolism and hyperaldosteronism, regardless of Cushingoid clinical manifestations, AVS should be the gold standard for making a definitive diagnosis and for lateralizing functioning adrenocortical adenomas. However, the interpretation of AVS findings in these patients is also challenging, because the aldosterone to cortisol ratio, which was the most widely used measurement in clinical practice, was affected by the relative amount of cortisol and aldosterone secreted. To minimize misinterpretation, we also measured the concentrations of plasma epinephrine in both adrenal veins and inferior vena cava to enable adjustment in the present patient [20].

Aldosterone is synthesized in the ZG, the outermost zone of the adrenal cortex of humans, whereas glucocorticoids (cortisol and corticosterone) are produced in the ZF, the mid zone [21]. Aldosterone-producing adenomas (APAs) and cortisol-producing adenomas (CPAs) represent benign tumors of the ZG and the ZF, respectively. Nishimoto et al. described the expression patterns of steroidogenic enzymes in normal adrenal glands, APAs and CPAs, and summarized the steroidogenic enzymes that are responsible for adrenocortical hormone production, namely CYP11B2, 11β-hydroxylase (CYP11B1), 3βHSD (upstream enzyme for both CYP11B2 and CYP11B1), and P450c17 (upstream enzyme for CYP11B1, but not for CYP11B2). In the ZG and APA, CYP11B2-positive cells co-express 3βHSD in the aldosterone synthetic pathway. In the ZF and CPA, CYP11B1-positive cells co-express 3βHSD and P450c17, both of which are enzymes upstream of CYP11B1 in the cortisol synthetic pathway [22, 23]. In this patient, we performed immunohistochemical analyses for CYP11B2, 3βHSD, and P450c17 and found that he had a right adenoma that autonomously produced aldosterone and a left adenoma that autonomously produced cortisol, consistent with the results of AVS. Immunohistochemistry has become an important tool for the diagnosis of adrenocortical pathological conditions, and is sometimes an indispensable step in confirming the diagnosis.

## Conclusions

In summary, we presented an extremely rare case of bilateral adrenal adenomas, the left of which secreted cortisol and the right of which secreted aldosterone, as determined by AVS and confirmed by histopathological evaluation of the resected surgical specimens, including immunohistochemical evaluation of steroidogenic enzymes. We suggested that patients with bilateral adrenal adenomas should be screened for both hyperaldosteronism and hypercortisolism to minimize misdiagnoses. AVS, with proper interpretations, is essential in such patients, and immunohistochemistry might become a widely used and powerful tool for identifying aldosterone/cortisol-producing lesions in close future.

## Additional file

Additional file 1: **Table S1.** The available case reports of with bilateral adrenocortical adenomas secreting cortisol or aldosterone independently. A/CPA, aldosterone- and cortisol-producing adenoma; APA, aldosterone-producing adenoma; ARR, aldosterone-to-renin ratio; AVS, adrenal venous sampling; BPA, Bilateral partial adrenalectomy; CPA, cortisol-producing adenoma; CS, Cushing’s syndrome; HE, hematoxylin–eosin stain; IHC, Immunohistochemical; LPA, Left partial adrenalectomy; LTA, Left total adrenalectomy; NM, not mentioned; PAC, plasma aldosterone concentration; PRA, plasma renin activity; RPA, Right partial adrenalectomy; RTA, Right total adrenalectomy. (DOCX 24 kb)

## Data Availability

Data sharing is not applicable to this article as no datasets were generated or analyzed during the current study.

## Abbreviations

24 h UFC: Twenty-four-hour urine free cortisol
3β-HSD: 3b-hydroxysteroid dehydrogenase
ACTH: Adrenocorticotropic hormone
APA: Aldosterone-producing adenoma
ARR: Aldosterone-to-renin ratio
AV: Adrenal vein
AVS: Adrenal venous sampling
CPA: Cortisol-producing adenoma
CS: Cushing’s syndrome
CT: Computed tomography
CYP11B1: 11β-hydroxylase
CYP11B2: 11beta-hydroxylase 2
HbA1c: glycosylated hemoglobin
IVC: Inferior vena cava
P450c17: 17alpha-hydroxylase1
PA: Primary aldosteronism
PAC: Plasma aldosterone concentration
PRA: Plasma renin activity
SCS: Subclinical Cushing’s syndrome
ZF: Zona fasciculate
ZG: Zona glomerulosa
ZR: Zona reticularis
