# Supplementary material for: Hypercortisolism and primary aldosteronism caused by bilateral adrenocortical adenomas: a case report
Source: BMC Endocr Disord. 2019 Jun 17;19:63. doi: 10.1186/s12902-019-0395-y (PMC6580498; doi:10.1186/s12902-019-0395-y)
Supplement: Supplementary file 1 — Table S1. The available case reports of with bilateral adrenocortical adenomas secreting cortisol or aldosterone independently. A/CPA, aldosterone- and cortisol-producing adenoma; APA, aldosterone-producing adenoma; ARR, aldosterone-to-renin ratio; AVS, adrenal venous sampling; BPA, Bilateral partial adrenalectomy; CPA, cortisol-producing adenoma; CS, Cushing’s syndrome; HE, hematoxylin–eosin stain; IHC, Immunohistochemical; LPA, Left partial adrenalectomy; LTA, Left total adrenalectomy; NM, not mentioned; PAC, plasma aldosterone concentration; PRA, plasma renin activity; RPA, Right partial adrenalectomy; RTA, Right total adrenalectomy. (DOCX 24 kb) [file 12902_2019_395_MOESM1_ESM.docx]

| Study ID | Country | Age | Gender | Hypertension | Hypokalemia | CS | PRA  (ng/ml.h) | PAC  (ng/dl) | ARR  (ng/dl:ng/ml.) | AVS |
| --- | --- | --- | --- | --- | --- | --- | --- | --- | --- | --- |
|  |  |  |  |  |  |  |  |  |  |  |
| 1991 Nagae, A.^10^ | Japan | 55 | Female | Y | Y | Y | 1.33 | 24.2 | 18.2 | Y |
| 2008 Oki, K.^11^ | Japan | 50 | Male | Y | Y | N | 1.1 | 20.3 | 18.5 | Y |
| 2009 Onoda, N.^12^ | Japan | 43 | Female | Y | Y | Y | 1.5 | 30.5 | 20.3 | Y |
| 2011 Morimoto, R.^13^ | Japan | 54 | Female | Y | N | Y | 0.1 | 12.8 | 128 | Y |
| 2015 Seung-Eun Lee.^14^ | Korea | 31 | Female | Y | Y | N | 0.76 | 24.4 | 32.11 | Y |

Supplementary Table 1. The available case reports of with bilateral adrenocortical adenomas secreting cortisol or aldosterone independently.

CS, Cushing’s syndrome; PRA, plasma renin activity; PAC, plasma aldosterone concentration; ARR, aldosterone-to-renin ratio; AVS, adrenal venous sampling;

| Study ID | Lesions size (cm) | | Diagnosis | | Operation | Cut surface | | HE | IHC |
| --- | --- | --- | --- | --- | --- | --- | --- | --- | --- |
|  | Right | Left | Right | Left |  | Right | Left |  |  |
| 1991 Nagae, A.^10^ | 0.9 | 2.4, 0.8 | APA | APA,CPA | RPA, LTA | golden yellow | golden yellow, dark brown | Y | N |
| 2008 Oki, K.^11^ | 1.1 | 1.0 | A/CPA | APA | RTA, LPA | NM | golden yellow | Y | Y |
| 2009 Onoda, N.^12^ | 3.0 | 2.4 | CPA | A/CPA | BPA | yellow | golden yellow | Y | Y |
| 2011 Morimoto, R.^13^ | 2.5, 2.1 | 1.6, 1.9 | APA,CPA | APA | RPA, LTA | NM | NM | Y | Y |
| 2015 Seung-Eun Lee.^14^ | 1.6 | 2 | CPA | APA | RPA, LTA | bright yellow | brown | Y | N |

Supplementary Table 1. (Continued).

HE, Hematoxylin–eosin stain; IHC, Immunohistochemical; APA, aldosterone-producing adenoma; CPA, cortisol-producing adenoma; A/CPA, aldosterone- and cortisol-producing adenoma; RPA, Right partial adrenalectomy; LTA, Left total adrenalectomy; RTA, Right total adrenalectomy; LPA, Left partial adrenalectomy; BPA, Bilateral partial adrenalectomy; NM, not mentioned
